# Knowledge, Attitude, and Practice towards Glycemic Control and Its Associated Factors among Diabetes Mellitus Patients

**DOI:** 10.1155/2019/2593684

**Published:** 2019-04-08

**Authors:** Daniel Asmelash, Netsanet Abdu, Samson Tefera, Habtamu Wondifraw Baynes, Cherie Derbew

**Affiliations:** ^1^Department of Clinical Chemistry, School of Biomedical and Laboratory Sciences, College of Medicine and Health Sciences, University of Gondar, P.O. Box 196, Gondar, Ethiopia; ^2^Department of Medical Laboratory Sciences, School of Biomedical and Laboratory Sciences, College of Medicine and Health sciences, University of Gondar, Gondar, Ethiopia

## Abstract

**Background:**

Diabetes mellitus is a metabolic disorder of multiple etiologic factors characterized by chronic hyperglycemia with disturbance of carbohydrate metabolism. It can play the vital role in the cause of morbidity and mortality through continued clinical consequence. Therefore, good knowledge, attitude, and practices of glycemic control are necessary in promoting care, in enhancing better therapeutic outcomes, and in the prevention and management of diabetes complications.

**Methods:**

A cross-sectional study design was conducted to determine knowledge, attitude, and practice towards glycemic control and its associated factors. Diabetic patients who were attending the University of Gondar Hospital from March to May 2018 were included in the study. The data was collected using questionnaires, and individuals that can fulfill our inclusion criteria were selected by a simple random sampling technique. SPSS version 20 was used for descriptive and logistic regression analysis, and finally, the variables were summarized and presented using tables and graphs.

**Results:**

Of the total 403 participants, 216 (53.6%) were males and 176 (43.7%) were illiterate. Of the total, 250 (62%) had good knowledge, 271 (67.2%) had a good attitude, and 300 (74.4%) had good practice towards glycemic control. In multivariate logistic regression, occupational status and marital status were significantly associated with the knowledge of participants towards glycemic control. Occupational status, educational status, and marital status were significantly associated with attitude and practice towards glycemic control.

**Conclusion:**

More than half of the participants had good knowledge, attitude, and practice towards glycemic control. Occupational status and marital status were significantly associated with knowledge, attitude, and practice towards glycemic control.

## 1. Background

Diabetes mellitus (DM) is a group of metabolic disorders characterized by hyperglycemia. It is associated with abnormalities in carbohydrate, fat, and protein metabolism, which results in chronic complications, including microvascular, macrovascular, and neuropathic disorders [1]. DM is due to either the pancreas unable to produce insulin or the body cell which cannot respond to insulin [2].

Throughout the last twenty years, the incidence of diabetes has been raised intensively in many parts of the world [3]. Globally, an estimated 422 million adults are living with DM, according to the latest 2016 data from the World Health Organization (WHO). The number is projected to almost double by 2030. Increases in the overall diabetes prevalence rates largely reflect an increase in risk factors for type 2 DM, notably being overweight or obese [4].

In 2010, 12.1 million people were estimated to be living with diabetes in Africa, and this is projected to increase to 23.9 million by 2030 [5, 6]. In Ethiopia, the prevalence of diabetes was 3.5% in 2011, and the extrapolated prevalence in 2013 was 4.36%. It is also known that a large number of people remain undiagnosed, with an estimated number of undiagnosed cases reported to be 1.39 million people in 2013 [6, 7].

Regardless of the pathogenesis, uncontrolled diabetes or poor glycemic control is associated with chronic hyperglycemia, leading to the development of long-term microvascular, macrovascular, and neuropathic complications. According to the American Diabetes Association, the target for long-term glycemic control in patients with diabetes is glycated hemoglobin A1c (HbA1c) value of less than 7% [8]. Studies have shown that significant reduction in the mortality and morbidity occurs with the improved glycemic control. This may be due to a reduction in microvascular complications like low systemic inflammation, by the prevention of immune dysfunction and protection of the endothelium and of the mitochondrial ultrastructure and function [9].

Diabetic complications such as diabetic ketoacidosis, micro- and macrovascular diabetic complications, and their associated adverse outcomes are intimately related to suboptimal glycemic control in clinical practice. Each 1% reduction in the mean glycated hemoglobin (HbA1c) has been shown to be associated with a reduction in risk of 21% for deaths related to diabetes, 14% for myocardial infarction, and 37% for microvascular complications [10].

The management of DM largely depends on the patient's ability to do self-care in their daily lives, and therefore, patient education is always considered an essential element of DM management. Studies have shown that patients, who are knowledgeable about the DM self-care, have better long-term glycemic control [11]. Knowledge about glycemic control can help the people to understand the risk of diabetes and motivate them to seek proper treatment and care and to keep the disease under control [8].

Many studies have shown that control of hyperglycemia in diabetic patients can prevent or reduce the risks of diabetic complications. Better glycemic management of DM requires not only the prescription of an appropriate nutritional and pharmacological regime by the physician but also intensive education of the patient. Most studies have used measurements such as blood glucose level and knowledge, attitude, and practice (KAP) as the index of diabetes management [12].

Nowadays, people in developing countries like Ethiopia are suffering from chronic diseases, of which diabetes is the major one having a significant contribution to mortality and morbidity. Diabetes is a self-managed condition; therefore, knowledge, attitudes, and practices about glycemic control in DM patients can influence the overall treatment outcomes and the complications of the disease. Identification of knowledge, attitudes, and practices towards glycemic control would provide better insight for the development of preventive and treatment strategies for the patients.

## 2. Methods

### 2.1. Study Design, Study Period, and Study Population

A cross-sectional study was conducted to determine the level of KAP towards glycemic control and its associated factors among DM patients at the University of Gondar Hospital from March to June 2018. The study population was all diabetic mellitus patients who visited the University of Gondar Hospital during the study period and fulfilled the inclusion criteria.

### 2.2. Study Variables

The dependent variables were knowledge, attitude, and practice, and the independent variables were sex, age, ethnicity, educational status, occupational status, religion, marital status, and duration of diabetes mellitus.

### 2.3. Inclusion and Exclusion Criteria

All DM patients who volunteered to give information about their knowledge, attitude, and practice towards glycemic control were included in the study. Patients with mental health problems and hearing impairments and those patients who were unable to provide the appropriate information were excluded.

### 2.4. Sample Size and Sampling Technique

The required sample size was determined by using a single population proportion formula. Therefore, the proportion was taken at 50%, and the sample size calculation was made as the following proportion of the study with 95% of confidence intervals (CI) and 5% of margin error.

*n* = *z*^2^*p* (1 − *p*)/*w*^2^, where *n* = sample size, *p* = proportion (50%), *w* = margin error (5%), *z* = 1.96 confidence level, and *n* = 1.96^2(0.5(1 − 0.5))/(0.05)(0.05)^ = 384. By considering the 5% nonresponse rate, the sample size was 403 and a simple random sampling technique was used to select those study participants.

### 2.5. Assessment of Knowledge, Attitude, and Practice

Knowledge about glycemic control was assessed using 16 general questions which were considered to be known by diabetic patients like the importance of glycemic control, risk factors, and complications of poor glycemic control. Each response was scored as “1” for correct response and “0” for incorrect responses. Knowledge scores of individuals were calculated and summed up to give the total knowledge score. Participants who correctly responded to more than 50% of knowledge questions were considered as having adequate knowledge about glycemic control, whereas those who scored <50% were considered as having inadequate knowledge about glycemic control.

Similarly, 12 attitude- and 10 practice-related questions were asked, and the responses to each question were scored as “1” for correct response and “0” for incorrect responses. Participants who correctly responded more than 50% of attitude and practice assessing questions were considered as having good attitude and practice towards glycemic control, whereas those who scored ≤50% were considered as having a poor attitude towards glycemic control.

### 2.6. Data Collection Procedure

The data were collected by the structured questionnaire, which contains different items like sociodemographic and KAP towards glycemic controls.

The questionnaire was prepared, first in English, and then, it was translated into local language, Amharic, to collect the data. The questionnaire was prepared by investigators based on the variables and objectives of the study.

### 2.7. Data Analysis and Interpretation

After data collection, the response was coded and entered into the computer using EPI info data version 7 and the data was analyzed by using SPSS version 20. All independent variables with a *P* value less than 0.2 in the bivariate analysis were included in the multivariate models to identify factors associated with knowledge, attitude, and practice towards glycemic control. A *P* value less than 0.05 was considered as statistically significant. Frequencies and percentages were calculated for all variables, which are related to the objectives of the study. Besides, the relationships between knowledge, attitudes, and practice scores were examined using bivariate correlation analysis. The study result was presented by using tables and graphs and interpreted by using OR and *P* value.

### 2.8. Ethical Consideration

Ethical clearance was obtained from the research and ethics committee of the School of Biomedical and Laboratory Science, College of Medicine and Health Science, University of Gondar. The participants recruited to the study were informed about the objectives of the study, and their confidentiality was kept by using codes. Informed consent was obtained from each participant before the data collection.

## 3. Results

### 3.1. Sociodemographic Characteristics of the Study Participants

From a total of 403 participants, 216 (53.6%) were males. In the majority of the participants, 108 (26.8%) were farmers, 176 (43.7%) were illiterate, and 221 (54.8%) were within the age group of 46 years and above (Table 1).

### 3.2. Knowledge of Study Participants

Of all participants, 250 (62%) had good knowledge towards glycemic control with the knowledge mean score of 10.2 ± 4.33. In the majority of participants, 341 (84.6%) had good knowledge about the effect of extra salt intake, and 321 (79.7%) had knowledge on how to inject insulin medication. However, only 159 (39.5%) participants were known to have hereditary DM (Table 2).

### 3.3. Attitude of Study Participants

Out of 403 participants, 271 (67.2%) had a good attitude towards glycemic control with an attitude mean score of 7.28 ± 2.14. In the majority of participants, 373 (92.3%) believed that modern medication was better than traditional treatments for glycemic control and 288 (71.5%) of them believed that smoking can increase the complications of diabetes (Table 3). However, in less than half of participants, 189 (46.9%) believed fruits and vegetables are good for glycemic control.

### 3.4. Practices of Study Participants

Out of the study population, 300 (74.4%) had good practices towards glycemic control with a practice mean score of 6.6 ± 1.75. In less than half of participants, 176 (43.7%) had a good eye/foot care practice. However, in almost all participants, 399 (99%) had good medication adherence and 393 (97.5%) had checked their blood sugar at least once in the last three months (Table 4).

**Figure 1:**
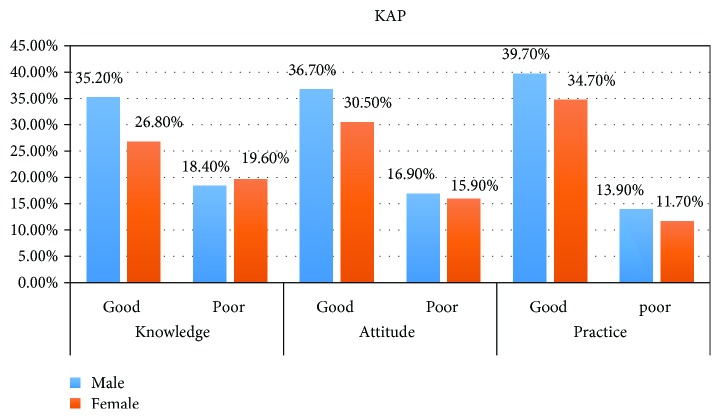
The level of KAP towards glycemic control by sex of DM patients at the University of Gondar Hospital, 2018.

In addition, we have tried to assess the correlation between knowledge, attitude, and practices of the study participants based on the Spearman correlation. Knowledge and attitude scores of the participants achieved a significant positive correlation (*r* = 0.681). Similarly, knowledge and practice scores of the participants had statically shown a significant positive correlation (*r* = 0.516). In addition, attitude and practice scores showed a positive correlation (*r* = 0.53) (Figure 1).

### 3.5. Factors Associated with Knowledge

In multivariate logistic regression, marital status and occupational status were significantly associated with knowledge towards glycemic control of diabetes (Table 5).

### 3.6. Factors Associated with Attitudes

In multivariable logistic analysis, marital status, occupational status, and educational status were significantly associated with the attitude of participants towards glycemic controls of diabetes (Table 6).

### 3.7. Factors Associated with Practices

In multivariate logistic regression, occupational status, educational status, and marital status of the participants were significantly associated with practices towards glycemic controls (Table 7).

## 4. Discussions

Out of 403 participants, 250 (62%) had a good knowledge. This finding was higher than the study done in Bale Town, Ethiopia (52.5%) [13]; Debre Tabor, Ethiopia (49%) [14]; Sudan (15%) [15]; Malaysia (41.9%) [16]; and UAE (33%) [11]. This may be because the study participants were hospital-based and they have better health education access. In contrast, this finding was lower when compared to the study done in Mekelle, Ethiopia (93.7%) [17], and in Assam University Clinic and Mother and Child Hospital Buraydah, India (71.9%) [18]. This might be due to the difference in health education, sample size, and access to sources of information like television, radio, and newspaper.

In this finding, more than half of participants, 58.3%, know the cause of DM; this finding was lower than the study done in rural Bangladesh (93%) [19]. This difference may be due to limited sources of information, inadequate involvements of media, and other concerned body on knowledge towards glycemic control. In this study, 49.6% of participants had responded they did not know any complication regarding DM. This finding was high when we compared it with a study done in India; 18% of the participants did not know the complications of DM [20]. This might be due to the higher literacy rate among study participants in India.

Of the participants, 62.3% were knowledgeable about the meaning of DM and 59.8% about the risk factors of DM. This study was higher than the study done in Bale, Ethiopia, in which 54.5% knew what DM means and 48% were able to identify the risk factors of DM [13], and in studies done in India, 50% of participants were knowledgeable about the meaning of DM and 54% were knowledgeable on the risk factors of DM [20]. This could be because our study was hospital-based and they had a health education program.

The current study showed that 271 (67.2%) had a good attitude about glycemic controls. This finding was higher than the study done in Bangladesh (18%) [19], Kenya (49%), and India (17.6%) [21, 22]. This might be that studies done in Kenya and India were from rural communities, but our study was hospital-based and they have better access to a health education program than rural communities.

Of all participants, 144 (35.7%) of them believed that the necessity was medication for controlling glucose with diet rather than diet alone. This finding was lower than the studies conducted in Pakistan (68%) [23]. This might be due to educational status and poor health education about the necessity of nutrition.

Among 403 participants, 74.4% showed good practice towards glycemic control. This finding was lower compared to the studies conducted in South Africa (99%) [24]. However, it was higher than the studies done in Harar, Ethiopia, in which 39.2% had good self-care practice [7]. This might be due to difference in sociodemographic and access to health education programs.

A total of 99% of participants had medication adherence, and 11.2% had a history of smoking. This finding was inconsistent with studies done in Addis Ababa, Ethiopia, where 97% adhered to prescribed medication and 12% of all respondents have the habit of smoking [25]. In addition, of the total, 260 (51%) had no daily exercise activity, which was inconsistent with studies done in Addis Ababa, Ethiopia (49%) [26].

The majority of participants, 393 (97.5%), had their blood sugar level checked for the last 3 months. This finding was higher than the study done in rural Bangladesh (47.5%) [19] and Pondicherry, India (78.8%), in which the participants had their blood sugar checked at least once in the last 3 months [27]. Because the current study was hospital-based, they might have access to health education programs, which can increase the awareness and practice of the DM patients towards glycemic control.

Less than half of participants, 176 (43.7), had a good eye/foot care practice. This study result was higher than the studies done in Iran, in which 33% [3] had a good eye/foot care practice. However, it was lower than the studies done in United Arab Emirates where 81.8% had a good foot care practice [11]. This might be due to difference in health beliefs, demographic characteristics, and diabetes education programs.

In this study, occupation and marital status were significantly associated with knowledge of participants using multivariate logistic regression. This finding was similar to the study done in Mekelle, Ethiopia [17]. Educational and occupational status showed a significant association with the practice towards glycemic control. This finding was similar to the study conducted at Nekemte, Ethiopia [28], and Addis Ababa, Ethiopia [25]. This is because participants who had higher educational status have more awareness about diabetes mellitus.

## 5. Conclusions

More than half of the participants had good knowledge, attitude, and practice towards glycemic controls. Occupational and educational status was the variable which remained to be significantly associated with knowledge towards glycemic control. In addition, occupation, education, and marital status were significantly associated with attitude and practice towards glycemic control.

### 5.1. Recommendations

A hospital-based intervention program should be implemented in order to improve the KAP of patients regarding glycemic control.

### 5.2. Limitation of the Study

The KAP question response of participants might be affected by both interviewers and recall bias. In addition, the result of this study cannot be inferred to other populations in the country because KAP might be greatly influenced by sociodemographic factors of the population.

## Figures and Tables

**Table 1:** Sociodemographic characteristics of the study participants, at the University of Gondar Hospital, 2018.

Variables	Categories	Frequency	Percent (%)
Sex	Male	216	53.6
	Female	187	46.4

Age	18-25 years	52	12.9
	26-35 years	66	16.4
	36-45 years	64	15.9
	≥46 years	221	54.8

Level of education	Unable to read and write	176	43.7
	Primary	71	17.6
	High school	73	18.1
	College/university and postgraduate	83	20.6

Marital status	Married	267	66.3
	Divorced	31	7.7
	Widowed	43	10.7
	Single/never married	62	15.4

Occupation	Government employed	76	18.9
	Unemployed	84	20.8
	Merchant	82	20.3
	Day laborers	15	3.7
	Farmers	108	26.8
	Others	38	9.4

**Table 2:** Knowledge assessment towards glycemic controls among DM patients at the University of Gondar referral hospital 2018.

Knowledge assessment items	Correct response *n* (%)	Incorrect response *n* (%)
Definition of DM	251 (62.3)	152 (37.7)
What type of DM you had	252 (62.5)	151 (37.5)
Causes of DM	235 (58.3)	168 (41.7)
Type of medications used	341 (84.6)	62 (15.4)
What to do when you become hypoglycemic	337 (83.6)	66 (16.4)
How to inject insulin	321 (79.7)	82 (20.3)
Is that DM hereditary?	159 (39.5)	244 (60.5)
What does lipidemic/obesity/hypertension mean?	277 (68.7)	126 (31.3)
Risk factors of DM	241 (59.8)	162 (40.2)
How can DM be detected?	184 (45.7)	219 (54.3)
Could DM affect other organs?	134 (43.9)	221 (56.1)
Can complications occur due to DM?	203 (50.4)	200 (49.6)
Effect of regular exercise on DM	344 (85.4)	59 (14.6)
Effect of extra salt intake on DM	362 (89.8)	41 (10.2)
Effect of extra sugar intake on DM	253 (62.8)	150 (37.2)
Effect of smoking on DM	275 (68.2)	128 (31.8)

**Table 3:** Attitude assessment towards glycemic controls among DM patients at the University of Gondar referral hospital 2018.

Attitude assessment	Correct response *n* (%)	Incorrect response *n* (%)
Do you think glycemic control is necessary for DM?	282 (94.8)	21 (5.2)
Do you think regular exercise can help to control DM?	348 (86.4)	55 (13.6)
Do you think smoking causes poor glycemic control?	288 (71.5)	115 (28.5)
Do you think blood pressure control is necessary for glycemic control?	288 (71.5)	115 (28.5)
Do you think glycemic control prolonged life expectancy?	263 (65.3)	140 (34.7)
Do you think that alternative treatments are good?	111 (27.5)	292 (72.5)
Do you believe good blood sugar control is important for DM?	360 (89.3)	43 (10.7)
Do you think diet alone glycemic control is better than medication with diet glycemic control?	144 (35.7)	259 (64.3)
Do you believe fruits and vegetables are good for glycemic control?	189 (46.9)	214 (53.1)
Do you think alcohol can increase the complication of DM?	362 (89.8)	41 (10.2)
Do you think insulin (metformin) drug has harmful effects to the organs of the body?	166 (41.2)	237 (58.8)
Do you think traditional treatments are better than modern medicines for DM?	372 (92.3)	31 (7.2)

**Table 4:** Practice assessments towards glycemic controls among DM patients at the University of Gondar referral hospital 2018.

Practice assessment	Yes *n* (%)	No *n* (%)
Eat vegetables daily	183 (45.4)	220 (54.6)
Daily physical exercise	197 (48.9)	260 (51.1)
Medication/treatment adherence	399 (99)	4 (1)
Control/maintain body weight	228 (56.6)	175 (43.4)
Regular blood sugar checkup	393 (97.5)	10 (2.5)
Cigarette smoking	45 (11.2)	358 (88.8)
Extra sugar/salt on your regular diet	74 (18.3)	325 (80.6)
Do you drink alcohol?	199 (49.4)	204 (50.6)
Do you eat food on time?	390 (96.8)	13 (3.2)
Eye/foot care	176 (43.7)	227 (56.3)

**Table 5:** Bivariant and multivariable analysis of factors associated with knowledge towards glycemic controls on DM patients at the University of Gondar referral hospital, 2018.

Variables	Knowledgeable		COR 95% CI	AOR 95% CI	*P* value
	Good	Poor			
*Sex*					
Male	142 (56.8%)	74 (48.4%)	1.00	1.00	
Female	108 (43.2%)	79 (51.6%)	0.712 (0.476, 1.067)	0.95 (0.59, 1.53)	0.86^#^
*Age group*					
18-25	34 (13.6%)	18 (11.8%)	1.00	1.00	
26-35	43 (17.2%)	23 (15.0%)	0.990 (0.461, 2.124)	0.91 (0.34, 2.4)	0.85^#^
36-45	43 (17.2%)	21 (13.7%)	1.084 (0.500, 2.350)	1.16 (0.42, 3.2)	0.76^#^
≥46	130 (52.0%)	91 (59.5%)	0.756 (0.402, 1.421)	0.92 (0.36, 2.36)	0.87^#^
*Marital status*					
Married	176 (43.7%)	91 (59.5%)	1.00	1.00	
Divorced	14 (5.6%)	17 (11.1%)	0.426 (0.20, 1.903)	0.485 (0.216, 1.087)	0.079^#^
Single	45 (18%)	17 (11.1%)	1.369 (0.742, 2.526)	1.628 (0.840, 3.15)	0.015^∗^
Widowed	15 (6%)	28 (18.3%)	0.277 (0.141, 0.545)	0.257 (0.112, 0.590)	0.001^∗^
*Occupations*					
Unemployed	48 (19.2%)	36 (23.5%)		1.00	
Merchant	49 (19.6%)	33 (21.6%)	1.114 (0.600, 2.065)	0.707 (0.341, 1.464)	0.35^#^
Government employed	73 (29.2%)	3 (2.0%)	18.250 (5.319, 62.614)	9.772 (2.650, 36.0)	0.001^∗^
Day laborers	7 (2.8%)	8 (5.2%)	0.656 (0.218, 1.977)	0.366 (0.112, 1.199)	0.097^#^
Farmer	49 (19.6%)	59 (38.6%)	0.623 (0.351, 1.107)	0.352 (0.176, 0.707)	0.003^∗^

^∗^Significantly associated. ^#^Not significantly associated.

**Table 6:** Bivariable and multivariable analysis of factors associated with attitude towards glycemic controls on DM patients at the University of Gondar referral hospital, 2018.

Variables	Attitude		COR 95% CI	AOR 95% CI	*P* value
	Good	Poor			
*Age*					
18-25	35 (12.9%)	17 (12.9%)	1.00	1.00	
26-35	45 (16.6%)	21 (15.9%)	1.041 (0.478, 2.264)	0.701 (0.24, 2.01)	0.508^#^
36-45	44 (16.2%)	20 (15.2%)	1.069 (0.488, 2.341)	0.813 (0.27, 2.36)	0.704^#^
≥46	147 (54.2%)	74 (56.1%)	0.965 (0.507, 1.836)	0.64 (2.34, 1.75)	0.38^#^
*Marital status*					
Married	193 (71.2%)	74 (56.1%)	1.00	1.00	1
Divorced	17 (6.3%)	14 (10.6%)	0.466 (0.21, 0.992)	0.588 (0.249, 1.386)	0.225^#^
Single	40 (14.8%)	22 (16.7%)	0.697 (0.388, 1.252)	0.339 (159, 0.721)	0.005^∗^
Widowed	21 (7.7%)	22 (16.7%)	0.366 (0.190, 0.705)	3.287 (1.725, 6.262)	0.002^∗^
*Occupations*					
Unemployed	52 (19.2%)	32 (24.2%)	1.00	1.00	1
Merchant	55 (20.3%)	27 (20.5%)	1.254 (0.663, 2.371)	0.46 (0.2, 1.05)	0.083^#^
Government employed	73 (26.9%)	3 (2.3%)	14.974 (4.352, 51.525)	0.86 (0.177, 4.209)	0.001^∗^
Day laborers	8 (3.0%)	7 (5.3%)	0.703 (0.233, 2.125)	0.39 (0.107, 1.47)	0.242^#^
Farmer	60 (22.1%)	48 (36.4%)	0.769 (0.430, 1.376)	0.325 (0.15, 0.698)	0.004^∗^
Others	23 (8.5%)	15 (11.4%)	0.944 (0.430, 2.070)	0.25 (0.09, 0.7)	0.008^∗^
*Education*					
Unable to read/write	78 (28.8%)	98 (74.2%)	1.00	1.00	
Primary school	49 (18.1%)	22 (16.7%)	2.798 (1.560, 5.020)	3.287 (1.725, 6.262)	0.001^∗^
High school	66 (24.4%)	7 (5.3%)	11.846 (5.145, 27.274)	13.562 (5.414, 33.97)	0.001^∗^
College and above	78 (28.8%)	5 (3.8%)	19.600 (7.56, 50.773)	20.615 (5.901, 72.02)	0.001^∗^

^∗^Significantly associated. ^#^Not significantly associated.

**Table 7:** Bivariable and multivariable analysis of factors associated with practices towards glycemic controls on DM patients at the University referral hospital of Gondar, 2018.

Variables	Category	Practice		COR 95% CI	AOR 95% CI	*P* value
		Good	Poor			
Sex	Male	160 (53.3%)	56 (54.4%)	1.00	1.00	
	Female	140 (46.7%)	47 (45.6%)	1.043 (0.665, 1.634)	1.23 (0.47, 1.23)	0.114^#^

Age	18-25	43 (14.3%)	9 (8.7%)	1.00	100	
	26-35	49 (16.3%)	17 (16.5%)	0.603 (0.2441, 0.493)	0.45 (0.32, 4.1)	0.42
	36-45	48 (16.0%)	16 (15.5%)	0.628 (0.252, 1.567)	0.78 (0.35, 1.68)	0.46
	≥46	160 (53.3%)	61 (59.2%)	0.549 (0.253, 1.194)	0.85 (0.35, 1.78)	0.22

Marital status	Married	204 (68.0%)	63 (61.2%)	1.00	1.00	
	Divorced	22 (7.3%)	9 (8.7%)	0.755 (0.331, 1.723)	2.121 (0.853, 5.275)	0.331^#^
	Single	47 (15.7%)	15 (14.6%)	0.968 (0.507, 1.847)	1.410 (0.450, 4.419)	0.322^#^
	Widowed	27 (9.0%)	16 (15.5%)	0.521 (0.264, 1.028)	0.735 (0.219, 2.469)	0.066^#^

Occupational status	Unemployed	63 (21.0%)	21 (20.4%)	1.00	1.00	
	Merchant	69 (23.0%)	13 (12.6%)	1.769 (0.818, 3.827)	0.861 (0.344, 2.157)	0.894^#^
	Government employed	69 (23.0%)	7 (6.8%)	3.286 (1.308, 8.254)	0.478 (0.121, 1.885)	0.00^∗^
	Day laborers	11 (3.7%)	4 (3.9%)	0.917 (0.264, 3.188)	0.476 (0.115, 1.973)	0.69^#^
	Farmer	64 (21.3%)	44 (48.7%)	0.485 (0.259, 0.906)	0.228 (0.102, 0.510)	0.001^∗^
	Others	24 (8%)	14 (13.5%)	0.571 (0.251, 1.302)	0.164 (0.057, 0.474)	0.003^∗^

Educational status	Unable to read/write	107 (35.7%)	69 (67.0%)	1.00	1.00	
	Primary	53 (17.7%)	18 (17.5%)	1.899 (1.027, 3.510)	1.929 (0.975, 3.815)	0.049^∗^
	High school	66 (22.0%)	7 (6.8%)	6.080 (2.636, 14.02)	7.07 (2.792, 17.93)	0.00^∗^
	College/university	74 (24.7%)	9 (8.7%)	5.302 (2.492, 11.28)	5.78 (1.890, 17.71)	0.002^∗^

^∗^Significantly associated. ^#^Not significantly associated.

## Data Availability

The data generated or analyzed during this study were included in this published article.

## Abbreviations

AOR: Adjusted odds ratio
COR: Crude odds ratio
CI: Confidence interval
DM: Diabetes mellitus
HbA1c: Glycated hemoglobin A1c
WHO: World Health Organization
KAP: Knowledge, attitude, and practice.
